# Mutation of nucleotides around the +1 position of type 3 polymerase III promoters: The effect on transcriptional activity and start site usage

**DOI:** 10.1080/21541264.2017.1322170

**Published:** 2017-06-09

**Authors:** Zongliang Gao, Alex Harwig, Ben Berkhout, Elena Herrera-Carrillo

**Affiliations:** Laboratory of Experimental Virology, Department of Medical Microbiology, Center for Infection and immunity Amsterdam (CINIMA), Academic Medical Center, University of Amsterdam, Amsterdam, The Netherlands

**Keywords:** +1 position, initiation accuracy, small RNA, transcriptional efficiency, type 3 Pol III promoter

## Abstract

Type 3 RNA polymerase III (Pol III) promoters are widely used for the expression of small RNAs such as short hairpin RNA and guide RNA in the popular RNAi and CRISPR-Cas gene regulation systems. Although it is generally believed that type 3 Pol III promoters use a defined transcription start site (+1 position), most man-made promoter constructs contain local sequence alterations of which the impact on transcription efficiency and initiation accuracy is not known. For three human type 3 Pol III promoters (7SK, U6, and H1), we demonstrated that the nucleotides around the +1 position affect both the transcriptional efficiency and start site selection. Human 7SK and U6 promoters with A or G at the +1 position efficiently produced small RNAs with a precise +1 start site. The human H1 promoter with +1A or G also efficiently produced small RNAs but from multiple start sites in the −3/−1 window. These results provide new insights for the design of vectors for accurate expression of designed small RNAs for research and therapeutic purposes.

## Introduction

Small RNAs play regulatory roles in diverse intracellular processes in both eukaryotes and prokaryotes. In addition, small RNA molecules have been exploited as powerful tools for biomedical research and therapeutic purposes. This includes small interfering RNA (siRNA) or short hairpin RNA (shRNA) in RNA interference (RNAi) experiments, guide RNA (gRNA) in clustered regularly interspaced palindromic repeats (CRISPR)-Cas applications and RNA molecules such as aptamers, ribozymes, and antisense RNA.^1-7^ Type 3 polymerase III (Pol III) promoter-based vectors have been developed to express these small RNAs inside cells because of high transcriptional activity and defined transcription initiation and termination sites, thus allowing the production of very precise RNA molecules.

The accurate expression of designed small RNAs is important for the proper and specific execution of their function. For instance, for shRNA molecules, the sequence precision is critical because the stem-loop structure is processed by the Dicer endonuclease that measures ∼21-nucleotides (nt) from the 5′ terminus and length variation at this end will yield a different RNA duplex with different silencing activity and specificity.^8,9^ The recently described Dicer-independent AgoshRNA molecules are directly loaded into Argonaute 2 (Ago2) for processing and subsequent mRNA silencing,^10^ but the identity of the 5′ end nt was shown to be critical for AgoshRNA silencing activity and specificity.^11,12^ The prokaryotic CRISPR-Cas9 system has been adapted for gene editing in mammalian cells and requires a gRNA to guide the Cas9 endonuclease for site-specific genome editing.^2,13,14^ The synthesis of a precise gRNA molecule is extremely important because the 5′ end 20-nt of the gRNA plays a role in target site recognition.^13,15^ The newly discovered Cpf1 endonuclease uses a single ∼43-nt CRISPR-derived RNA (crRNA) as guide and the 5′ end was shown to be pivotal for function.^16^ Other RNA molecules like aptamers and ribozymes may also need precise 5′ and 3′ ends to adopt the active RNA conformation, which can be critical for target binding and/or catalytic target RNA cleavage.^3,6^ Thus, small effector RNAs require a specific 5′ end to ensure proper function.

Pol III promoters are efficient in transcribing high levels of small non-coding RNAs and promoters such as 7SK, U6, and H1 are therefore widely used.^2,13, 17-19^ Another advantage of such “gene-external” promoters is that all important sequence elements are positioned upstream of the +1 position, thus allowing the expression of almost any RNA sequence of interest. It is generally assumed that type 3 Pol III promoters start transcription precisely at the +1 position, which is defined by a specific distance from the TATA box, a major specificity determinant in these promoters.^17, 20-23^ A recent study on the mouse U6 promoter indicated that transcription starts most efficiently at the first A or G within the −1/+2 window.^24^ The importance of the identity of the +1 nt in different type 3 Pol III promoters has not been thoroughly surveyed. In this study, we focused on three human type 3 Pol III promoters: 7SK, U6, and H1. We demonstrated that nucleotides around the +1 position affect the efficiency of small RNA transcription and start site usage. This study provides important insights for efficient and precise small RNA expression in human cells.

## Results

### The 7SK promoter with +1A/G efficiently produces small RNA with a precise 5′ end

**Figure 1.**
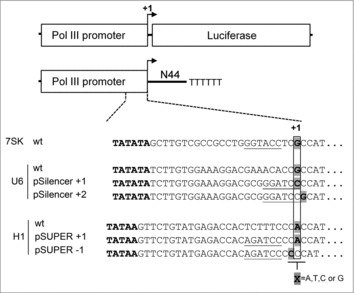
Schematic diagram of Pol III promoter based constructs. Pol III promoters (7SK, U6, and H1) were used to drive Firefly luciferase (Luc) expression and N44 transcription. The +1 position is the putative transcription start site defined by the TATA boxes (shown in bold). Partial wt promoter sequences containing TATA boxes and +1 nt are shown. The complete promoter sequences are provided in the supplemental material. For the U6 and H1 promoters, the wt sequences around +1 position were changed into useful restriction enzymes sites (underlined) to facilitate cloning. The X marked in gray was mutated into A, T, C, or G in both the Luc and N44 constructs.

The human 7SK promoter initiates transcription of the abundant 7SK nuclear RNA that represses Pol II transcription elongation by binding to the elongation factor P-TEFb.^25^ Due to the existence of a useful *Acc*65I restriction enzyme site in the wild type (wt) promoter sequence (GGTACC underlined in Fig. 1), this sequence was maintained in the popular psiRNA-7SK vector that was successfully used for shRNA expression.^26,27^ The putative start site (+1G) of the human 7SK RNA gene is located 24-nt downstream of the TATA box (marked in bold). A survey of the 7SK promoter sequence in 14 vertebrate species present in the NCBI database (http://www.ncbi.nlm.nih.gov/) indicated that all use +1G (results not shown).

**Figure 2.**
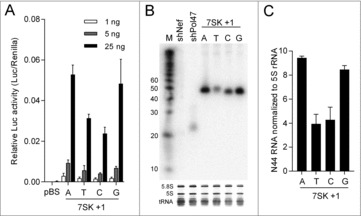
Luc activity and N44 transcription of 7SK +1 variants. (A) Relative Luc activity of 7SK +1 variants. 7SK-Luc constructs were titrated (1, 5, and 25 ng) and 1 ng of Renilla plasmid was co-transfected into HEK 293T cells. The pBS plasmid was used as negative control. Two days post-transfection, cells were lysed and Dual-Luciferase assays were performed. The ratio of Luc to Renilla was calculated as the relative Luc activity. Three independent transfections were performed. Error bars represent the standard deviations, *n* = 3. (B) Northern blotting of N44 RNA. Total RNA from 7SK-N44 transfected HEK 293T cells was isolated and analyzed by Northern blotting. The short hairpin constructs shNef and shPol47 were used as negative and positive control, respectively. The size (in nt) of RNA marker M is indicated. The rRNAs and tRNAs were stained with ethidium bromide as loading control. (C) Quantification of N44 RNA in (B) in two independent experiments. Error bars indicate standard errors of the mean, *n* = 2.

To examine whether the identity of the +1 nt affects 7SK promoter activity, we made all four possible +1 variants in two reporter constructs, 7SK-Luciferase (7SK-Luc) and 7SK-N44, a manmade sequence that produces a 44-nt transcript that lacks stable RNA structure (Fig. 1). The former allows the expression of the Firefly luciferase reporter.^28^ The latter was designed to probe the small ∼44-nt transcript products by Northern blotting. We first used the set of +1 variants to drive Luc expression in transfected HEK293T cells. A previous study indicated that functional mRNAs can be produced by Pol III.^29^ The 7SK-Luc constructs were titrated (1, 5, and 25 ng), and 1 ng of Renilla luciferase plasmid was co-transfected to control for transfection efficiency. Two days post-transfection, luciferase activity was measured and the ratio of Firefly to Renilla luciferase was calculated to represent the Luc expression level. The relative Luc activity of the +1 variants is plotted in Fig. 2A. The pBluescript SK (pBS) plasmid was used as negative control, the pGL3 plasmid in which Luc is transcribed by the strong SV40 Pol II promoter served as positive control that produced 20-fold higher Luc levels than the 7SK promoter (results not shown). The 7SK promoter with +1A and G induced similar Luc activity, more than 2-fold higher than the activity obtained for the +1T and C variants. Thus, it seems that the 7SK promoters with +1A or G are most active, although it cannot formally be excluded that the transcripts differ in stability or translational efficiency. These Luc results should be treated with caution as Pol III transcripts are uncapped and not polyadenylated and thus not optimally suited for Luc translation.

To distinguish between transcription versus post-transcriptional effects, we next used the 7SK +1 variants to transcribe the small unstructured N44 sequence (Fig. 1). Total cellular RNA was extracted at 48 h post-transfection of HEK293T cells, and Northern blotting was performed to detect the N44 transcript with the Pol47 probe. As expected, no transcript was detected for the negative control shNef, and the shPol47 construct produced a small ∼21-nt RNA (Fig. 2B). Transcripts of ∼44-nt were detected for the N44 constructs, and quantification of the signals showed the same pattern as observed in the Luc assays: The +1A and G variants were of comparable strength and 2-fold more active than +1T and C (Fig. 2B and quantification in Fig. 2C). Combining these results, we conclude that the 7SK promoter needs a purine at the +1 position to ensure efficient transcription.

**Figure 3.**
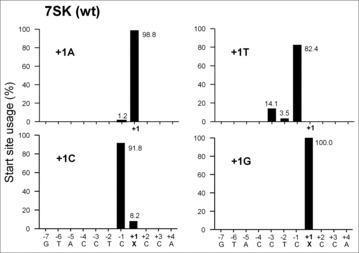
Start site usage of 7SK +1 variants. Start site mapping by GeneScan analysis. Total RNA from 7SK-N44 transfected HEK 293T cells was reversed transcribed using a FAM labeled primer and analyzed by GeneScan. The GeneScan output was corrected with Rox 500 Size Standard and analyzed by GeneMapper. The signals representing the relative start site usage of each construct was quantitated and the relative start site usage of the 7SK +1 variants is shown. The mutated nucleotide is marked as X.

We next wanted to investigate whether nucleotide changes at the +1 position also affect the transcription start site selection. 5′ rapid amplification of cDNA ends (5′ RACE) is a highly sensitive method for mapping the 5′ end of individual RNAs and was used for mapping Pol III transcription start sites. We previously mapped the transcription start site profile of the H1 promoter in the pSUPER construct.^30^ However, 5′ RACE is laborious and is not very quantitative and we obtained strange results for some constructs. We therefore set out to use fluorescent primer extension (FPE) based GeneScan analysis. This method provides precise sizing and quantitative information of fluorescently labeled DNA fragments and has been used successfully for quantitative mapping of transcription start sites.^31,32^ A limitation of the FPE method is that it does not discriminate between the incorporation of templated or non-templated nucleotides. Total RNA from 7SK-N44 transfected cells was reverse transcribed using a FAM-labeled primer complementary to the 3′ end of the N44 sequence, and the resulting cDNAs were subjected to GeneScan analysis. The relative frequency of start site usage was determined and distinct transcription start site profiles were apparent (Fig. 3). Transcripts start at +1 for the G construct. For the A construct, nearly all (98.8%) of the transcripts start at +1, but a minority (1.2%) start at −1. The predominant start site moves to the −1 position for the T and C constructs. Thus, only 7SK promoters with A and in particular G at the +1 position will start precisely at this position.

### The U6 promoter with +1A/G efficiently produces small RNA by precise +1 transcription

The human U6 promoter controls the transcription of the U6 small nuclear RNA (snRNA), which is an essential component of the spliceosome.^33^ The transcription start site (+1G) of the human U6 snRNA is located 24-nt downstream of the TATA box (Fig. 1). U6 promoter sequences of 23 eukaryotic species in the NCBI database all encode +1G (results not shown), suggesting that the sequence at this position is important for function. This +1G has been included in U6 promoter-based vector systems,^21,22,34^ but the local sequence was altered in the commonly used pSilencer vector to create a *BamHI* restriction enzyme site that facilitates cloning (GGATCC underlined in Fig. 1). Interestingly, the protocol provided by the pSilencer supplier Thermo Fisher Scientific suggests that +2, instead of +1, acts as the start site of transcription. To explore the effect of the +1 and +2 positions on U6 promoter activity, three sets of promoter variants were made: +1 variants in the context of the wt U6 promoter and +1 and +2 variants in the pSilencer context (Fig. 1).

**Figure 4.**
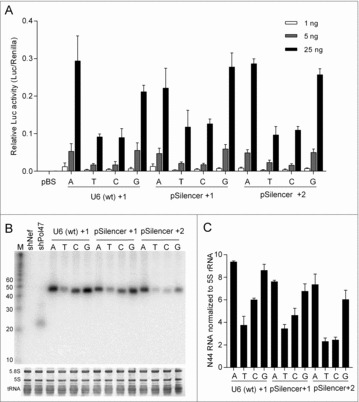
Luc activity and N44 transcription of U6 variants. (A) Relative Luc activity of three sets of U6 variants. U6-Luc variants were transfected into HEK 293T cells and the Dual-luciferase assays were performed as in Fig. 2B. Error bars indicate standard errors of the mean, *n* = 3. (B) Northern blotting of U6-N44 constructs. The shNef and shPol47 were used as control and the rRNAs and tRNAs served as loading control. (C) Quantification of N44 RNA from (B). Error bars represent standard errors of the mean, *n* = 2.

As described for the 7SK promoter, we used these U6 variants to drive Luc expression and N44 transcription. Consistent patterns were observed in the Luc assays for all three sets of U6 promoter constructs: The variants with A or G at the +1 or +2 position are stronger than the corresponding T and C constructs (Fig. 4A). Northern blotting of the N44 transcripts revealed a similar trend: the A or G constructs produce more N44 transcripts than the C and especially the T constructs (Fig. 4B, quantification in Fig. 4C). Thus, we conclude that a purine (A or G) around the +1 position is required for optimal U6 promoter activity.

**Figure 5.**
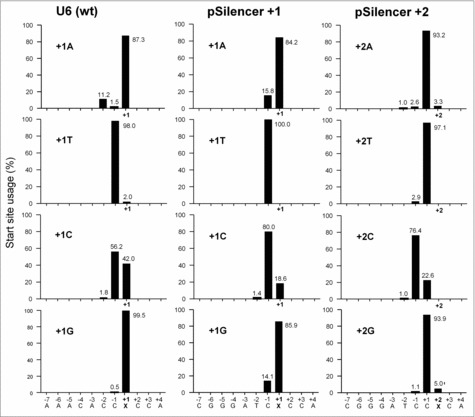
Start site usage of U6 variants. Total RNA was analyzed from cells transfected with U6-N44 constructs with +1 or +2 variation and subjected to GeneScan analysis. The relative start site usage of each construct was calculated as in Fig. 3. X marks the mutated position.

Next, we used GeneScan to map the transcription start sites (Fig. 5). The start site profile of the wt U6 set resembles that of the pSilencer +1 variants: Transcription starts predominantly at +1 when an A or G is located at this position, but the start site moves partially or completely to the −1 position for the C and T constructs, respectively. When the +2 position is mutated in the pSilencer context, little effect on +1 initiation was scored for the A, T, and G constructs, although a minority of the A/G transcripts starts at the +2 position. Strikingly, the +2C variant showed a profound move to −1C as the start site. To summarize, +1 nt identity is important for the U6 promoters and A or G yield an efficient start precisely at this position.

### Multiple transcription initiation sites used by the H1 promoter

The H1 promoter controls the transcription of H1 RNA, the RNA component of the nuclear RNase P enzyme.^23^ The putative start site (+1A) of the human H1 RNA gene is located 26-nt downstream of the TATA box. The H1 promoter sequence of 18 eukaryotes in the NCBI database shows variable +1 nt identity (5 × A, 2 × T, 2 × C, 9 × G). The H1 promoter therefore may offer flexibility for shRNA design as +1 nt alteration does not seem to affect the level of shRNA expression and gene silencing.^34^ Those H1 promoter properties differ significantly from those discussed for 7SK and U6.

**Figure 6.**
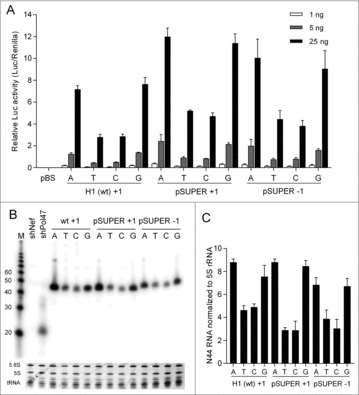
Luc activity and N44 transcription of H1 variants. (A) Relative Luc activity of the H1-Luc variants. The transfection experiments and the Dual-luciferase assays were performed as in Fig. 2B. Error bars represent standard errors of the mean, *n* = 3. (B) Northern blotting of N44 RNA made by the three sets of H1-N44 variants. The shNef and shPol47 served as control and the loading control was indicated by stained rRNAs and tRNAs. (C) Quantification of N44 RNA as in (B). Error bars represent standard errors of the mean, *n* = 2.

**Figure 7.**
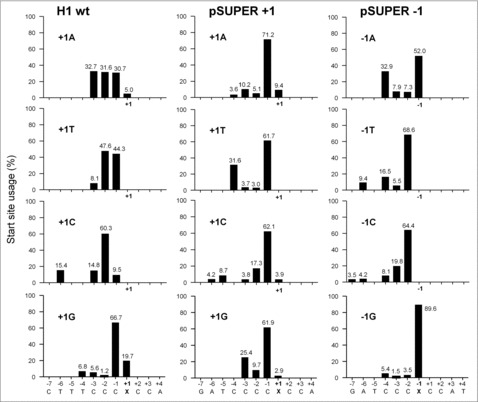
Start site usage of H1 variants. Total cellular RNA from H1-N44 constructs with −1 or +1 variation was subjected to GeneScan. The relative start site usage was determined as described in Fig. 3. X marks the mutated position.

The wt H1 promoter sequence in the frequently used pSUPER vector was altered to create a *BgIII* restriction enzyme site for easy cloning purposes (AGATCT, which was changed to AGATCC upon insertion of the Luc and N44 sequences, underlined in Fig. 1). To examine whether +1 nt identity affects H1 promoter transcription, we analyzed +1 nt variation in the wt H1 and pSUPER context. Luc reporter assays (Fig. 6A) and the N44 transcript level on Northern blot (Fig. 6B and quantification in Fig. 6C) showed a similar pattern as observed for the 7SK and U6 promoters: The A and G constructs are approximately 2-fold stronger than the T and C variants. GeneScan analysis indicated, much to our surprise, that the wt H1 promoter and all +1 variants use multiple start sites, mostly within the −3/−1 window (Fig. 7). However, −1A became the predominant start site in all pSUPER +1 variants, likely induced by the insertion of the restriction enzyme site. We therefore generated an additional set of −1 variants of pSUPER and observed good Luc activity (Fig. 6A) and moderate N44 transcript levels for the −1A and G constructs (Fig. 6B and C). Position −1 is the predominant, but not the exclusive start site for the −1A and G constructs (Fig. 7). The −1G constructs achieves 89.6% precision for transcription initiating at this −1 position. Multiple start sites were also observed for the −1T and C constructs, but with −2 as the predominant start site. Taken together, we conclude that the H1 promoter requires a purine around position +1 for efficient transcription. This promoter tends to start transcription from multiple sites between the −3/−1 positions.

### Novel insight used to create improved H1-gRNA cassettes

**Figure 8.**
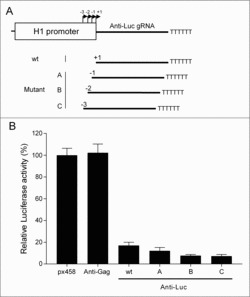
Design of optimal gRNAs using H1 initiation knowledge. (A) The H1 promoter drives anti-Luc gRNA expression. The H1 promoter tends to initiate transcription in the −3/−1 region instead of the wt +1 position. The wt anti-Luc gRNA was stepwise moved 1–3 nt upstream, creating the A–C mutants. (B) Luc knockdown activity of the anti-Luc gRNAs. An equal molar amount of px458 construct was co-transfected with 200 ng Luc plasmid and 2 ng Renilla control plasmid. The ratio of Luc to Renilla was calculated as the relative Luc activity. The empty px458 vector was used as control to set the Luc activity at 100%. The anti-Gag is an unrelated gRNA construct. Error bars indicate standard errors of the mean, *n* = 3.

The finding of variable transcription initiation sites, in particular, for the H1 promoter, may have important implications for the design of small RNA constructs, e.g., shRNA vectors for RNAi experiments or gRNA vectors for popular CRISPR-Cas9 applications. To test this, we designed different H1-gRNA constructs directed against the same target sequence in the Firefly luciferase reporter. The wt H1 promoter places an anti-Luc gRNA exactly at the predicted +1 start site (Fig. 8). As we just learned that H1 transcription will actually initiate in the scattered −3/−1 region, this may add unwanted 5′-end nucleotide to the gRNA. To correct for this, we gradually moved the gRNA sequence upstream by 1, 2, or 3 positions over the +1 position (Fig. 8A, mutants A–C). We realize that this is a rather complex modification, e.g., changing the initiation efficiency by a change of the +1 nt and changing the actual length of the gRNA. Nevertheless, we scored the silencing activity of these four constructs upon co-transfection with the target Firefly luciferase plasmid and a Renilla control plasmid (Fig. 8B). Increased silencing activity was scored for all three mutants compared with wt, especially for the B and C variants. The wt construct will transcribe a gRNA with a variable 5′-end extension of 1–3 nt, which is suboptimal for gRNA efficiency,^35^ and the A–C constructs gradually shorten this 5′-end tail, which coincides with the increased silencing activity. This pilot result demonstrates that the design of optimal gRNAs should incorporate the new information on transcription initiation that is disclosed in this study.

## Discussion

Numerous small regulatory RNAs exist in both eukaryotes and prokaryotes and several RNA-based systems have been exploited to regulate gene expression in mammalian cells. Expression of these small regulatory RNAs is usually achieved by type 3 Pol III promoters. It is commonly believed that type 3 Pol III promoters start transcription at the +1 position, which exhibits a certain nucleotide preference. For example, both the human 7SK and U6 promoters start transcription at +1G, 24-nt downstream of the respective TATA box.^20,22^ In fact, these two promoters use the same TATA box sequence (Fig. 1). In this study, we demonstrated that these two commonly used promoters require A or G at or around +1 for efficient and precise transcription.

This new insight on the +1A/G start site provides guidelines for the efficient and precise production of small RNAs using these two promoters. The default criterion for designing shRNAs and gRNAs in RNAi and CRISPR-Cas9 applications with the U6 promoter is preserving a G at the +1 position, but our current findings suggest that RNA molecules can be designed with either +1A or G as the 5′ terminal nucleotide. This finding also provides important insight for the design of Dicer-independent AgoshRNA molecules. Unlike canonical shRNA, the AgoshRNA molecule requires Ago2 for both processing and the subsequent mRNA cleavage reaction.^36^ The MID domain of Ago2 exhibits higher binding affinity for RNAs with a 5′-terminal A or U than C or G.^12,37^ Combined with the new initiation knowledge for 7SK and U6 promoters, +1A should be adopted for 7SK/U6-AgoshRNA constructs to ensure high expression of active molecules. Indeed, pilot experiments indicate that the AgoshRNA design with +1A is most active (unpublished results).

The U6-based pSilencer +2A and G constructs exhibited minor +2 start site usage, suggesting that this vector, which was specifically designed for shRNA expression, may add an additional nucleotide that might comprise its function. Interestingly, this characteristic of the human U6 promoter disagrees with results described for the mouse U6 promoter, according to which the first A or G present in the −1/+2 window is the predominant transcription start site.^24^ These two U6 promoters have limited sequence identity (52.9% in the complete promoter segment),^38^ which may relate to these differences.

For the human H1 promoter, a purine around the +1 position ensures efficient transcription. However and unlike the 7SK and U6 promoters, the H1 promoter tends to start from multiple sites within the −3/−1 window. In fact, this upstream shift brings the distance between the TATA box and start site within the range observed for the 7SK and U6 promoters. Thus, the H1 promoter produces transcripts with significant 5′ end variation, which may jeopardize the function of the encoded small RNAs. Inspired by this knowledge, we purposely designed anti-Luc gRNA constructs by realigning the small gRNA with the upstream transcription initiation sites (−3/−1). We measured increased anti-Luc activity, especially for the −2/−3 construct compared with the standard +1 construct, highlighting the importance of start site knowledge. A complicating factor is that different H1 constructs are in use, and commercial sources have generated modified H1 constructs with convenient restriction sites for cloning purposes.^24,26^ We demonstrate that these local sequence changes have an impact on the position of the transcription initiation site, thus one should be careful in designing shRNA/gRNA constructs. We previously demonstrated that H1 promoter mutants exhibit a similar activity profile in different cell lines.^30^

A previous study indicated that a yeast internal Pol III promoter prefers purines at the transcription initiation site.^39^ We demonstrated that this is also true for the 7SK and U6 promoters, but not the H1 promoter that starts predominantly at pyrimidines. This suggests that purine preference is not a universal property of Pol III promoters. In all cases, the presence of purine-rich sequences around the +1 position stimulates transcription. Pol II transcription also shows a preferential requirement for +1 purine.^40,41^ The transcription initiation mechanism of Pol II and III was shown to be similar. The Pol II subunits Rpb4 and Rpb7 are important for transcription initiation and functional homologs exist in Pol III.^42^ Besides, these two polymerases form similar subcomplexes that function in initiation and start site selection.^42^ This similarity in polymerase composition may relate to the common purine preference.

The three Pol III promoters analyzed in this study have been widely used for synthesis of small RNAs, but some reports indicated that the U6 promoter can also drive the expression of a Luc reporter gene, resulting in a translation-competent mRNA.^28,43^ Here, we demonstrate that all three promoters can mediate Luc expression to variable degrees (H1>>U6>7SK). This suggests significant differences in capability of these promoters to transcribe long translation-competent transcripts. Strikingly, the H1 Pol III promoter produces 3–4 times more Luc protein than the regular SV40 Pol II promoter (results not shown). These results are rather surprising as the extended Luc mRNA contains multiple T stretches that should act as Pol III termination signals. More research is needed to analyze these events, but the initial data may suggest another reason why the H1 promoter is not ideal for small RNA synthesis.

## Materials and methods

### Plasmid construction

The psiRNA-h7SK hygro G1 (Invivogen), pSilencer 2.0-U6 (Ambion), and pSUPER (OligoEngine) vectors that contain different human Pol III promoters were used. The actual sequences of the 7SK, U6 and H1 promoters used in the study are provided as Supplemental material. The N44 sequence (5′-ACCATGGAAGTGAAGGGGCAGTAGTAATATACCGGTGATATCTT-3′) with nucleotide variation around +1 was inserted into these three vectors.^26^ The Pol III promoters with nucleotide variation around +1 were digested with appropriate restriction enzyme sites and inserted into the pGL3 vector (Firefly luciferase reporter) to replace the SV40 promoter, resulting in modified pGL3 constructs with Pol III promoter driving Luc expression. For wt U6 and H1 based constructs, fusion PCR was performed to create promoter-N44 fragments, which were subsequently ligated into the appropriate vectors.

To construct the anti-Luc CRISPR-Cas9 system, the CRISPR plasmid px458 (Addgene plasmid #48138) was used as backbone. We designed gRNAs targeting HIV Gag (5′-GCTACCATAATGATGCAAAG-3′) and the Firefly luciferase reporter (5′- GTGAACTTCCCGCCGCCGTT-3′). All H1-gRNAs were synthesized by Integrated DNA technology (IDT) and cloned into px458 by Gibson cloning according to the protocol (New England Biolabs). All constructs were verified by sequencing using BigDye Terminator v1.1 Cycle Sequencing Kit (ABI).

### Cell culture

Human embryonic kidney (HEK) 293T cells were cultured in Dulbecco's modified Eagle's medium (Life Technologies, Invitrogen, Carlsbad, CA, USA) supplemented with 10% fetal calf serum (FCS), penicillin (100 U/ml) and streptomycin (100 μg/ml). The cells were trypsinized and seeded one day before transfection.

### Luciferase assays

0.5 ml DMEM/10% FCS with 1.5 × 10^5^ cells was seeded in 24-well plates. Pol III promoter-based pGL3 plasmids were titrated (1, 5, or 25 ng), and 1 ng of Renilla luciferase plasmid (pRL) was co-transfected into HEK293T cells with Lipofectamine 2000 (Invitrogen) according to the manufacturer's instructions. For measuring the anti-Luc activity of the gRNAs, equal amounts (200 ng) of px458 plasmid were co-transfected with 200 ng pGL3-control and 2 ng pRL plasmid. Two days post-transfection, luciferase activity was measured with the Dual-Luciferase Reporter Assay System (Promega, Madison, WI, USA) according to the manufacturer's protocol. The ratio of Firefly to Renilla luciferase was used for normalization of the transfection efficiency. Three independent transfections were performed, each in duplicate. The resulting six values were corrected for between session variations as described previously.^44^

### N44 transcript detection by Northern blotting

Northern blotting was performed as described previously.^26,36^ Briefly, 1.5 × 10^6^ HEK293T cells per 25 cm^3^ flask were transfected with equimolar quantities (5 μg) of N44 constructs using lipofectamine 2000 (Invitrogen). Total cellular RNA was extracted two days post-transfection with the mirVana miRNA isolation kit (Ambion). The RNA concentration was measured with NanoDrop 2000 (Thermo Fisher Scientific). 5 μg of total RNA was electrophoresed in a 15% denaturing polyacrylamide gel (Precast Novex TBU gel, Life Technologies). [γ-^32^P]-labeled decade RNA marker (Life Technologies) was run along-side for size estimation. To check for equal sample loading, the gel was stained in 2 μg/ml ethidium bromide for 20 minutes and visualized under UV light. The RNA in the gel was electro-transferred to a positively charged nylon membrane (Boehringer Mannheim, GmbH) and cross-linked to the membrane using UV light (1200 μJ × 100). Locked nucleic acid (LNA) oligonucleotides (Pol47: 5′-ATTACTACTGCCCCTTCAC-3′) were 5′ end-labeled with the kinaseMax kit (Ambion) in the presence of 1 μl [γ-^32^P]-ATP (0.37 MBq/μl, PerkinElmer). Sephadex G-25 spin columns (Amersham Biosciences) were used to remove the unincorporated nucleotides. The membrane was incubated in 10 ml ULTRAhyb hybridization buffer (Ambion) at 42°C for 30 minutes, after which the labeled LNA oligonucleotides were added. After overnight hybridization at 42°C, the blot was washed twice for 5 minutes at 42°C with 2 × SSC/0.1% SDS and twice for 5 minutes at 42°C with 0.1 × SSC/0.1% SDS. The signals were captured by the Typhoon FLA 9500 (GE Healthcare Life Sciences) and quantified using ImageQuant. Two independent transfections and Northern blottings were performed.

### Mapping transcription start sites by fluorescent primer extension

5 μg of total cellular RNA of N44-transfected cells was reverse transcribed with the First-Strand cDNA Synthesis Kit (ThermoScript RT-PCR system). The 6-FAM labeled primer (5′-GATATCACCGGTATATTA-3′) complementary to the 3′ end of the N44 RNA sequence was used for primer extension. After incubation with RNase H, the cDNA was concentrated by ethanol precipitation and mixed with 1.5 μl Rox 500 Size Standard and run on an ABI PRISM 3010 XL Genetic Analyzer (Applied Biosystems) using default parameters. All data were analyzed using GeneMapper® software v4.0 (Applied Biosystems) and then manually corrected based on fragment size to generate the N44 transcription start site profile.

## Supplementary Material

1322170_Supplemental_Material.docx
