# Supplementary material for: Mutation of nucleotides around the +1 position of type 3 polymerase III promoters: The effect on transcriptional activity and start site usage
Source: Transcription. 2017 Jun 9;8(5):275–87. doi: 10.1080/21541264.2017.1322170 (PMC5703244; doi:10.1080/21541264.2017.1322170)
Supplement: 1322170_Supplemental_Material.docx [file ktrn-08-05-1322170-s001.docx]

**DNA sequences of Pol III promoters**

7SK promoter (wt):

CTGCAGTATTTAGCATGCCCCACCCATCTGCAAGGCATTCTGGATAGTGTCAAAACAGCCGGAAATCAAGTCCGTTTATCTCAAACTTTAGCATTTTGGGAATAAATGATATTTGCTATGCTGGTTAAATTAGATTTTAGTTAAATTTCCTGCTGAAGCTCTAGTACGATAAGTAACTTGACCTAAGTGTAAAGTTGAGATTTCCTTCAGGTTTATATAGCTTGTGCGCCGCCTGGGTACCTC

U6 promoter (wt):

GAGGGCCTATTTCCCATGATTCCTTCATATTTGCATATACGATACAAGGCTGTTAGAGAGATAATTGGAATTAATTTGACTGTAAACACAAAGATATTAGTACAAAATACGTGACGTAGAAAGTAATAATTTCTTGGGTAGTTTGCAGTTTTAAAATTATGTTTTAAAATGGACTATCATATGCTTACCGTAACTTGAAAGTATTTCGATTTCTTGGCTTTATATATCTTGTGGAAAGGACGAAACACC

U6 promoter in pSilencer 2.0-U6 vector:

GAGGGCCTATTTCCCATGATTCCTTCATATTTGCATATACGATACAAGGCTGTTAGAGAGATAATTAGAATTAATTTGACTGTAAACACAAAGATATTAGTACAAAATACGTGACGTAGAAAGTAATAATTTCTTGGGTAGTTTGCAGTTTTAAAATTATGTTTTAAAATGGACTATCATATGCTTACCGTAACTTGAAAGTATTTCGATTTCTTGGGTTTATATATCTTGTGGAAAGGACGCGGGATC

H1 promoter (wt):

GGAATTCGAACGCTGACGTCATCAACCCGCTCCAAGGAATCGCGGGCCCAGTGTCACTAGGCGGGAACACCCAGCGCGCGTGCGCCCTGGCAGGAAGATGGCTGTGAGGGACAGGGGAGTGGCGCCCTGCAATATTTGCATGTCGCTATGTGTTCTGGGAAATCACCATAAACGTGAAATGTCTTTGGATTTGGGAATCTTATAAGTTCTGTATGAGACCACTCTTTCCC

H1 promoter in pSUPER vector:

GGAATTCGAACGCTGACGTCATCAACCCGCTCCAAGGAATCGCGGGCCCAGTGTCACTAGGCGGGAACACCCAGCGCGCGTGCGCCCTGGCAGGAAGATGGCTGTGAGGGACAGGGGAGTGGCGCCCTGCAATATTTGCATGTCGCTATGTGTTCTGGGAAATCACCATAAACGTGAAATGTCTTTGGATTTGGGAATCTTATAAGTTCTGTATGAGACCACAGATCCCC
